# Insights into the Government’s Role in Food System Policy Making: Improving Access to Healthy, Local Food Alongside Other Priorities

**DOI:** 10.3390/ijerph9114103

**Published:** 2012-11-12

**Authors:** Jessica Wegener, Kim D. Raine, Rhona M. Hanning

**Affiliations:** 1 School of Nutrition, Ryerson University, Toronto, Ontario M5B 2K3, Canada; 2 Centre for Health Promotion Studies, School of Public Health, University of Alberta, Edmonton, Alberta T6G 1C9, Canada; Email: kim.raine@ualberta.ca; 3 School of Public Health and Health Systems, University of Waterloo, Waterloo, Ontario N2L 3G1, Canada; Email: rhanning@uwaterloo.ca

**Keywords:** food policy, community food security, public health, government, land use planning, food access

## Abstract

Government actors have an important role to play in creating healthy public policies and supportive environments to facilitate access to safe, affordable, nutritious food. The purpose of this research was to examine Waterloo Region (Ontario, Canada) as a case study for “*what works*” with respect to facilitating access to healthy, local food through regional food system policy making. Policy and planning approaches were explored through multi-sectoral perspectives of: (a) the development and adoption of food policies as part of the comprehensive planning process; (b) barriers to food system planning; and (c) the role and motivation of the Region’s public health and planning departments in food system policy making. Forty-seven in-depth interviews with decision makers, experts in public health and planning, and local food system stakeholders provided rich insight into strategic government actions, as well as the local and historical context within which food system policies were developed. Grounded theory methods were used to identify key overarching themes including: “strategic positioning”, “partnerships” and “knowledge transfer” and related sub-themes (“aligned agendas”, “issue framing”, “visioning” and “legitimacy”). A conceptual framework to illustrate the process and features of food system policy making is presented and can be used as a starting point to engage multi-sectoral stakeholders in plans and actions to facilitate access to healthy food.

*I think that Government is a positive actor in society*…*I think Government has a positive role to play on a fiscal and policy side in society and the question for me becomes* *“Where can we pull on those levers to have the greatest impact on society at a reasonable cost*?”

(*Regional Councilor, 2009*)

## 1. Introduction

There has been a growing interest in linking food system policies and land use planning practices to healthier diets and healthier communities [1,2,3,4,5,6]. Little is known however about the process of food system policy making or the impact of planning and policy decisions in shaping local food systems and supportive community food environments, including opportunities for healthy food access. In 2009, Waterloo Region (Ontario, Canada) adopted a new Regional Official Plan (ROP), a long-range community planning framework that includes a progressive commitment to support the regional food system through actions to facilitate access to healthy, local food [7]. The ROP’s food policies were established based on the idea that multiple health, environmental, and local economic benefits can be achieved through a strong and diverse regional food system. The policies include a series of targeted planning actions to: protect the Region’s agricultural land; permit a full range of agriculture- and farm-related uses on agricultural land (*i.e.*, to support farmer viability); allow for a mix of land uses, including food destinations within close proximity to each other to increase neighbourhood access to food; and permit temporary farmers’ markets and community and rooftop gardens.

In light of the progressive and prescriptive nature of the food planning policies, the purpose of this study was to examine Waterloo Region as a case study for “what works” with respect to improving access to healthy food as a key concern and priority for public health alongside other important government priorities. Early government interest in promoting the *regional* food system stemmed from the Public Health Department’s concern for the loss of regional farmland, the rising price of fuel and the impact of redundant trade on local farmer viability in the Region. There were also concerns that, despite the Region’s strong agricultural economy, individuals and groups in the community did not have access to food. Structural changes within the Health Department, including the establishment of a unique “Health Determinants Division”, allowed staff to move beyond their traditional focus on individual food security to a broader exploration of the factors and conditions that shape community food security. Specifically, government actors in Public Health became passionate champions in their efforts to ensure that *all* community residents obtain a safe, culturally acceptable, nutritionally adequate diet through a sustainable food system that maximizes community self-reliance and social justice [8].

From a community food security perspective, efforts to strengthen the regional food system can help to improve physical access to healthy, locally-grown food by increasing retail opportunities and distribution sites close to places where residents live and work. Similarly, supportive planning considerations can reduce the barriers to local food production, processing and distribution (on and off the farm) and help to foster a food system that: supports and optimizes community self-reliance; provides opportunities for all food system stakeholders to be engaged (including small-scale producers); and reduces the environmental impact of long distance food transport. In this way, government action to address community food security through regional food system policy making can contribute to a number of social, economic and environmental goals. However, despite the potential for governments to play a positive role in promoting local and regional food systems, there have been few published studies to date that explore the ways in which food system planning “ideas” reach the political agenda, are considered by government, and become adopted as part of official land use policies. This paper aims to address these gaps by exploring multi-sectoral perspectives of the role and motivations of new government actors—most notably the Region’s public health and planning departments—in advancing supportive policy and environmental changes to improve access to healthy food, alongside other important government priorities. Particular attention is paid to the local and historical contexts within which food system ideas were initiated by Public Health, shared with other government actors (agenda setting), developed into acceptable food policies by policy planners (formulation), and adopted by the Region’s decision makers (adoption). Key overarching themes and subthemes are explored and discussed in relation to the roles and motivations of new government actors in food system policy making. Lastly, a conceptual framework is presented as a summary of key features of the regional policy making process.

## 2. Methods

Following approval by the University of Waterloo Office of Research Ethics, in-depth, semi-structured interviews (n = 47) were conducted between October 2009 and May 2010 with decision makers (n = 15); regional and local staff experts in public health and planning (n = 16); and food system stakeholders (n = 16). Decision makers included 15 of the 16 appointed and elected regional councillors, and staff experts were senior- and project-level public health and planning professionals involved in the ROP consultation process. Food system stakeholders included local food producers, retailers and distributors, and representatives from other levels of government and community interest groups.

To increase the likelihood that the substantive and theoretical findings of this research would be meaningful within and outside Waterloo Region, a Project Advisory Committee (PAC) was established at the outset. The PAC—consisting of key regional staff and academic experts—informed early stages of recruitment and provided feedback on the interview guides. The establishment of the PAC was an intentional step to prepare for the in-depth interviews in that it sensitized the Principal Investigator (PI) to initial ideas to pursue, areas for questioning, and relevant probes.

The interview guides were adapted from previous policy work on the role of issue framing in the environmental tobacco smoke bylaw development process in Waterloo Region [9] however the questions were revised to reflect the food policy interests of the current study. The use of adapted interview guides (for decision makers and key informants) improves the credibility of the study and helps build the field of policy research by using a similar methodology as researchers working in other areas of public policy. Although the purpose of this study was not to conduct a formal analysis of the policy process, Howlett and Ramesh’s [10] policy cycle was used to narrow the focus of research questioning and to organize the subsequent coding and analysis of the data. In light of the recency of the ROP’s adoption, only the first three policy stages were considered, including agenda-setting, policy formulation, and decision making.

Participants were recruited through email and phone using contact details from regional and community Web Sites. Overall, the goal of recruitment was to obtain between 32–48 interviews in total, or enough to ensure theoretical saturation of themes (that is, no new or further relevant insights are being reached, and hence the concept is “saturated”). A rough estimate was developed based on the following sampling strategies and other comparable, peer-reviewed policy studies. Quota sampling was used to target the 16 elected Regional Councillors (decision makers) on Waterloo Regional Council. In Waterloo Region, Councillors represent seven Area Municipalities including three large urban cities and four rural townships. The goal was to obtain the perspectives of all 16 regional decision makers, including the Regional Chair. However, in anticipation of potential participation challenges (*i.e.*, time, availability, interest, *etc*.) non-proportional quota sampling was used to recruit a sample that would include the Regional Chair and at least one regional representative from each of the cities and townships (to achieve a relatively balanced sample of rural and urban perspectives). Expert sampling was used to elicit the perspectives of key regional staff experts, and local planning professionals. The sampling strategy involved putting together a sample of those individuals with known or verifiable experience and expertise. Specifically, the names of key planners, policy and public health experts were identified from regional planning and public health reports and confirmed by members of the PAC. Snowball sampling as well as local food networking sites were used to recruit regional food system stakeholders. Using contact information from government and community Web Sites, participants were sent an information letter that detailed the study’s objectives, purpose, and the potential impact of the research. The nature of the project was explained, confidentiality assured, and agreement to participate and to permit audio recording were confirmed by signed consent.

All recruited participants agreed to participate with the exception of one regional decision maker and one food system stakeholder (due to timing and scheduling difficulties). Interviews addressed the initiation, development and adoption of regional food policies and included an examination of the roles and motivations of key government and community actors in food system policy making.

The interviews were carried out by one researcher (JW), audio-recorded and transcribed verbatim. Grounded theory methods [11] guided data collection and analysis and the organization and coding of transcripts was done by hand as well as with QSR NVivo8^®^ software (Cambridge, MA. USA). As Pidgeon and Henwood [12] note, there are a number of shared techniques and strategies common to all versions of grounded theory. Of these, this study adopted the following: (1) open-coding schemes to capture detail, *etc*. (2) a theoretical sampling approach; (3) constant comparison (*i.e.*, comparing data instances, cases, and categories for similarities and differences); (4) written theoretical memos; (5) focused coding of selected core categories; and (6) conceptual models as a way to move analysis from description to theory.

Three phases, or different levels of coding and analysis, were used including initial, focused, and theoretical coding [11]. Through careful attending to the data, key themes emerged and it became possible to develop early ideas about theory in relation to regional food system policy making. The PI’s (JW) background and experiences (which ultimately shaped what was “attended to”) combined with the interpretation and constructions of participants’ own experiences of policy making, and resulted in the emergence of key concepts and ideas. Theoretical coding was used to identify possible relationships between categories and to move the analytic concepts from focused coding in a theoretical direction [11]. Throughout this stage, diagrammatic illustrations (*i.e.*, concept maps) were developed to portray the interactions and relationship between key concepts (overarching themes and subthemes). Through a series of iterations, a conceptual framework was developed to illustrate the key features of food policy making at the regional level (discussed below). In this way, key emergent themes were grounded in the data, and triangulation of sources, peer debriefing and member checks (*i.e.*, returning a sub-sample of transcripts to key informants to test the analytic categories and the interpretation of the findings) [13] helped to ensure credibility and enhanced the trustworthiness of the analysis. Previous publications by the authors include a detailed analysis of multi-sectoral perspectives of the key facilitators and barriers to food system policy making at individual and organizational levels [14] and the barriers to food system planning at the municipal level [15]. The findings presented below address the third key objective of a larger study which was to describe the role and motivation of new government actors, namely the Region’s public health and planning departments, in advancing plans to facilitate access to healthy food as an element of a more food secure community. An overview of the local and historical contexts (key contextual factors) is presented first as relevant background to the ROP consultation process, followed by the identification and exploration of this study’s key overarching themes and subthemes as they relate to the role and motivation of government in regional food policy making.

## 3. Results

### 3.1. Defining Government Roles and Motivations within a Local and Historical Context

In the Waterloo Region, the local and historical contexts were critical factors defining regional government’s participation in food system planning activity. As noted above, the motivations of key staff experts in the Region’s public health and planning departments had evolved over a decade prior to the development and adoption of food policies in the ROP. Key informants described public health staff as “creating a climate of change” through extensive community research and capacity building activities [13]. As early as 1999, staff experts began exploring issues of hunger and household food security and identified a number of factors affecting farmer viability and urban food access in the Region. Early ideas about the interconnectedness of these issues lead to a series of commissioned studies and reports on the state of the regional food system [16] and deepened the Department’s interest and commitment to developing a broader, more comprehensive approach to addressing food and agriculture concerns.

In 2003, the Planning Department began consultations with other regional departments on its growth management strategy. The Strategy was a response to trends in provincial planning, high forecasted population growth, and anticipated changes to the regional community. The Department’s policy experts were concerned about the Region’s ability to protect the area’s prime agricultural land from development interests, and recognized the need for a strong internal partner within government to support their plan for a proposed (and controversial) *Countryside Line* (the purpose of the Countryside Line is to contain future growth within the urban areas as a way to protect farmlands and sensitive natural areas from urban development). Despite a long history of departmental silos, Public Health’s established community networks and concern for rural health and farmer viability lead to a unique partnership with Planning and a shared interest in preserving the Region’s agricultural land. Public health staff engaged policy planners in discussions about community food security and helped them to see how government action to support the regional food system could help protect the rural countryside from sprawl (a key regional planning priority) and at the same time, protect the Region’s ability to produce and supply food sustainably in the future. It was within this context that proposed government actions to improve the conditions associated with community food security emerged and the upcoming *ROP *review—a process in which policy planners revise and develop long range planning policies for the Region—was recognized by Public Health as a window of opportunity through which to ensure wider government buy-in and adoption of supportive food policies. Thus, early *strategic* relationship building was identified as a key facilitating factor within the local and historical context and provides insight into departmental motivations to jointly address food and agriculture concerns as a new area of government interest.

### 3.2. Understanding Government Roles and Motivations in Food System Policy Making

Based on further analyses of multi-sectoral perspectives, three overarching themes (key themes) and four underlying themes (subthemes) were identified concerning the role and motivations of regional government actors in food system policy making and environmental change. Overarching themes included “strategic positioning”, “partnerships” and “knowledge transfer” and subthemes included “aligned agendas”; “issue framing”; “visioning” and “legitimacy”. Themes are explored through relevant case study examples and discussed in relation to the role and motivations of government actors in Waterloo Region.

#### 3.2.1. An Overview of Key Overarching Themes and Subthemes

Based on detailed accounts of personal experiences with the policy making process, it was clear that key informants were attempting to understand and make sense of their participation, role and contribution to supportive policy and environmental changes within the regional food system context. For Public Health and Planning (new food policy actors), their participation was described as “deliberate” and “purposeful” with the overall intent to influence broader regional changes. “*Strategic positioning*” was identified as the main overarching theme under which the other overarching and underlying themes (sub-themes) were positioned and understood within a policy making context. Specifically, the other key overarching themes (“partnerships” and “knowledge transfer”) were identified more generally as examples of the types of actions or approaches that were effectively and commonly used by government actors to advance supportive food policy and environmental changes. For example, in positioning a food system agenda, public health and planning actors established strategic “*partnerships*”, and used their community groups and networking channels to widely disseminate new food system ideas and policy options (“*knowledge transfer*”) within the Region.

Likewise, sub-themes were also identified under the key overarching theme of “strategic positioning” but were distinguished by their relationship to both the key overarching theme (strategic positioning), and the other overarching themes (partnerships and knowledge transfer). For example, sub-themes captured the most commonly identified examples of specific government actions that “worked” in Waterloo Region to advance regional food system policies and included “*aligned agendas*”, “*visioning*”, “*issue framing*” and “*legitimacy*”. An exploration of key overarching themes is explored below through relevant examples from multi-sectoral stakeholders, followed by a brief examination of the study’s sub-themes.

#### 3.2.2. Strategic Positioning—The Role and Motivation of Public Health in Identifying Key Areas of Influence and Strategic Assets

The best examples of “strategic positioning” were described in reference to the role and motivations of public health staff experts in general and the Department’s local food champion in particular. Key informant perspectives of Public Health’s actions to influence food policy considerations in the ROP provided an outsider’s view of “strategic positioning” while staff experts offered rich insider accounts of food policy change *within* government. Specifically, public health experts described the process as “walking a fine line” between working in the public’s interest as a regional department and “responding to what the politically-elected representatives want to see” in an Official Plan.

**Table 1:** Staff expert perspectives on the public health department’s key areas of influence and strategic assets (strategic positioning).

Area of Influence/Strategic Asset	Public Health Perspectives (Evidence of Strategic Positioning)
2008 Ontario Public Health Standards	“So under the standards that actually relate to healthy eating and active living, **our staff were influential** in ensuring that food systems policy got included in the standards.”
Regional planning	“I managed to capture [regional planner’s] attention who was the planner with the lead on the Regional Official Plan…Knowing he was a planner, and knowing the role of planners all along, **we had made efforts to get to know them**.”
Municipal planning	“So, we thought, ‘We’ve got to start getting our heads around land use policy’, right? Because **we think we have a toe in the door with planners to influence this.**”
Regional decision making	“I wouldn’t underestimate **the amount of resources that we put into influencing this**…Because it was something that the Region had direct control over, [so] **we put *more* effort into it because we had that sort of inside avenue to decision makers**.”
Community support	“I think what then happened is we realized **the other asset we had was huge community support, and huge partnerships with community players** …so we really turned to them.”
Regional policy options	“We had somebody who was trained as a land use planner at the time working in Public Health and **that had been a strategic and intentional thing because we had wanted to influence land use policy**.”
Regional planning (policy language)	“We became one of the stakeholders and were actually providing input into the Official Plan and were responding to comments that were coming from the public. **And we had an opportunity to review and comment on the various edits**.”

As illustrated by the series of relevant quotations in Table 1, an important feature of Public Health’s participation in food system change was the ability to identify and use key areas of influence and strategic assets (as a feature of strategic positioning) to influence the initiation, development and adoption of supportive food policies during the ROP review. The following staff expert perspectives offer insight into Public Health’s motivation for participating in food system policy making through examples of “*strategic positioning*”:

In addition to identifying key areas of influence and strategic assets, a second effective feature of Public Health’s participation in food system change was the Department’s ability to strategize across all organizational levels. Specifically, project-level staff in Public Health identified and built relationships with key policy planners (*i.e.*, those concerned about agriculture land preservation); while management, and senior-level staff officials built capacity for change at higher levels. Despite noted hierarchy within the Region’s organizational structure, staff experts described their approach to increasing support for food policy considerations among key decision makers and senior-level planners as “strategic” in nature. The significance of political strategizing within a regional organization is best captured by the following senior-level health perspective:

“*At some point it did become a senior-level project…Things weren’t communicated and they couldn’t be…Because you can’t talk about this too much because you run the risk of others seeing your strategy and if others see your strategy, they have a strategy against it.*”

(*Public Health Official, 2009*)

#### 3.2.3. Strategic Positioning—The Role and Motivation of Regional Planning in Establishing Strategic Internal and External Partnerships

Examples of Regional Planning’s use of “strategic positioning” were also recognized and discussed by key informants. Specifically, Planning was seen as playing a strategic role in obtaining internal and external support for the proposed *Countryside Line *and for urban intensification plans to increaseneighbourhood-level food access. In both instances, regional planners were limited in their capacity to act without the support of each of the Region’s seven Area Municipalities (the Region is classified as a two-tier municipality in the Ontario framework. Matters of regional importance and scale (e.g., regional land use planning, public health, transit) are planned and managed by regional government while all other matters of a community or neighbourhood character are the responsibility of area municipalities [15]). Strategically, policy planners recognized the need for area municipal (local) governments as critical external partners for the successful implementation of their proposed plans for the Region. The strategic nature of Planning’s approach to regional change is captured by a senior-level policy planner:

“*About seventy percent of the things we had to do weren*’*t ours to do*.*So what you had to do was to get other people to do them for you*, *to buy into it*, *and then adapt their capital programs*, *their work program*, *to do the things that were important to us*, *not necessarily important to them*.”

(*Regional Planning Expert*, *2009*)

The Region’s need to position a regional agenda between provincial and municipal levels of government was described as a unique contextual challenge for policy planners. Thus, an Area Municipal Working Group was established to: strengthen the Region’s relationship with the municipalities, secure the necessary buy-in from external partners, and move the Region forward on plans for urban intensification and agricultural land protection. While the lack of community interest and support for the *Countryside Line* presented an early regional set-back, it was seen as a window of opportunity for Public Health. Strategically, by reaching out to their established agriculture and food networks, Public Health could help increase community support for Planning and use this to leverage supportive food policy considerations in the ROP. The use of community partnerships (*i.e.*, an external asset) to transfer trust *internally *(“knowledge transfer”) further exemplifies Public Health’s use of “strategic positioning” as an effective approach to advancing a supportive food policy agenda. The value of transferring trust through internal and external partnerships, as a feature of “strategic positioning”, is described by a senior-level public health expert,

“*And so we collaborated. And the Planning Department got such rich*, *rich input and they were so delighted that I think that was probably a watershed that forged the partnership because they saw how we could be useful to them*…*Because we had a history with *[*the agricultural community*], *and we had trust with them*, *and we could actually transfer trust to the Planning Department*…”

(*Public Health Expert*, *2009*)

For new government actors, the overarching theme of “strategic positioning” highlights the importance of political astuteness and ongoing monitoring of the decision making environment. Further, the identification of key areas of influence and strategic assets, the ability to strategize across all levels within a regional organization, and the establishment of critical internal and external partnerships were found to be important features of “strategic positioning” and shown to advance a mutually-benefiting food system agenda. The importance of “knowledge transfer” (*i.e.*, the sharing of ideas and potential policy options and the transferring of trust) through internal and external networking channels and partnerships is also captured by these examples as an effective way in which government actors facilitate the consideration and adoption of new policy ideas.

#### 3.2.4. Aligned Agendas, Visioning, and Issue Framing: Sub-themes and Features of Strategic Positioning

The key overarching theme of “strategic positioning” was discussed in relation to the roles and motivations of new policy actors. Related to this, key informants also identified the effective role of government actors in aligning regional agendas (“*aligned agendas*”), “*visioning*” and “*issue framing*” as critical features of strategic positioning. These were identified as sub-themes and are explored as specific examples of multi-sectoral stakeholder perspectives of effective government action towards food policy and environmental change. “*Legitimacy*” was also identified as an important sub-theme and is discussed in the following section.

##### “Aligned Agendas”: Strategic Positioning through Partnerships and Knowledge Transfer

By raising concerns about community food security through established networks and partnerships, Public Health’s senior-level food champion was described as strategically aligning a food systems agenda with the Planning Department’s direction on agricultural protection. Likewise, at the corporate leadership level, department heads and senior leaders in planning and public health were also strategizing and negotiating ways to align a health agenda with other regional priorities. The following senior-level perspectives offer relevant insight into the value of “aligned agendas” as an effective approach to increasing departmental credibility and the acceptance of new food system ideas in early stages of policy development:

“*We recognized fairly quickly that the Medical Officer of Health got a lot more credibility than what the Director of Planning got*.*And so we used that to advance the combined interests of our two departments*.”

(*Policy Expert*, *2009*)

“*At the Corporate Leadership table*, *we thought strategically*, *we already knew we wanted to have a countryside line*, *a transit corridor*, *and intensification*, *and we knew that including a health argument would be a helpful thing to paint the picture of what we were trying to achieve*.”

(*Public Health Official*, *2009*)

With respect to the role of government actors in food policy and environmental change, this finding offers insight into the *strategic* nature of staff efforts to align departmental policy agendas and provides evidence of effective action to increase organizational capacity. In light of Waterloo Region Council’s interest in greater inter-departmental collaboration, the alignment of Health and Planning agendas was strongly supported by regional decision makers and resulted in the necessary approval and adoption of food policies in the ROP.

“Aligned agendas” (a sub-theme and feature of “strategic positioning”) also provided a thematic link between the other overarching themes: “partnerships” and “knowledge transfer”. Specifically, Public Health influenced regional policy considerations and political opinion by sharing critical perspectives, insight and evidence with other government actors and by strategically aligning their health agenda with other government interests and regional priorities through a strategic partnership with Planning. As illustrated by the following senior-level perspective of “what works”, collaborative internal government partnerships and the alignment of departmental agendas were key features of an effective, adopted approach to advancing regional food policy and environmental changes to improve healthy food access in this case study.

“*They *[*Regional Council*] *knew that there was going to be a lot of debate around the Countryside Line*…[*So*] *if you can line up more partners that actually support your perspective*, *it makes your case stronger*.*So it was in Planning*’*s interest to continually align Health with what they were trying to achieve*.”

(*Public Health Official*, *2009*)

##### “Visioning”: A Strategic Exercise in Knowledge Transfer

The use of “visioning” emerged as an important sub-theme and a second critical feature of “strategic positioning”. Visioning was described by multi-sectoral stakeholders as an effective—yet “soft”—approach used by government actors to influence social norms, values and practices. Specifically, senior policy experts described food policy making (*i.e.*, within the official planning process) as a “visioning exercise” and a way to “nudge” or “push people in the direction they would probably go”. Thus, the vision set out in the ROP’s food policies and accompanying preamble was an important way to *strategically* transfer new plans for urban intensification and agricultural land preservation to the community as part of the Region’s effort to improve community food security (*i.e.*, a secure and sustainable local food system, including the protection of agricultural land *and* better access to healthy food). According to policy experts, it was anticipated that a *vision *to strengthen the regional food system would engage area municipalities in food-related issues, lead to supportive planning considerations, and create opportunities for public-private partnerships. Further, and as noted by the quotation below, there was also the expectation that some community residents *might be influenced *to think more about their food purchasing/procurement behaviour. Thus, the value of the Region’s use of “visioning” through the inclusion of food policies and a supportive food system preamble in the ROP (*i.e.*, a tool for the “transfer” and promotion of new food ideas) as a way to affect social change at the local level is captured by a senior policy planner:

“*At least by putting it *[*food system planning*] *in the Official Plan*, *it has elevated it to the point that it will be part of the public discussions*…*Sometimes moving society in a direction is just prodding them along*, *it*’*s not solid regulation*.”

(*Regional Policy Planner*, *2009*)

The use of visioning in this example also provides insight into Planning’s level of willing engagement, or *participation,* in addressing food access concerns. In contrast to the use of regulatory power, efforts to promote the *regional* food system through policies and actions in the ROP shows the potential for governments to support healthy, *local* food initiatives alongside other regional priorities while minimizing public concern over the interference in market-driven activity.

##### “Issue Framing”: Appealing Strategically to Others

Participants described several examples where the strategic framing of food and agriculture issues helped secure the necessary support for a new policy direction. One of the most notable examples of issue framing was Planning’s ability to reframe early draft food policies with the support of Public Health as a way to effectively appeal to the interests of other key government actors. Initially, within the Region’s early plans for urban intensification, the draft food policies included municipal directives regarding the size and location of food stores at the neighbourhood-level (a feature of complete, mixed-use communities). However, municipal planners opposed the draft policies and questioned the Region’s authority over local level food decisions. To minimize municipal concerns, regional policy planners revised the policies by strategically positioning neighbourhood-level food access alongside Public Health’s ideas about community food security and by adopting a food *systems* issue frame. By diverting attention away from commercial interests, the ROP’s focus on access to healthy, *local* food and a strong and diverse regional food system was an effective and strategic way to appeal to internal and external partners as captured by the following:

“*We realized that by changing the focus to more of a food systems approach*, *it just clarified what it was the Region was trying to do and it meshed well with a lot of other goals in our Plan*…*And people started to see that by framing it the way we did*, *and promoting access to local food*, *that we were very much in line with what the Region was all about traditionally*.”

(*Policy Planner*, *2009*)

Another example of issue framing, as a feature of strategic positioning, was Public Health’s ability to strategically frame the issue of urban sprawl as a loss of rural “*foodland” *and a threat to community food security. Staff attracted public interest, appealed to decision makers, and formed a strategic early partnership with Planning by raising their concerns about the Region’s ability to produce food sustainably in the future. In addition, public health staff used the idea of neighbourhood “walkability” to increase planners’ awareness and action toward the reduction of food deserts and other food access barriers. For example, community data on residents’ preference for food stores as a walking destination was used to appeal to Planning’s interest in intensification and efforts to reduce automobile dependency. By framing the problem of food access alongside other important regional priorities, Public Health captured political attention for food-related issues and was invited to inform policy ideas and changes during the ROP review. These examples offer relevant insight into government actors’ use of issue framing as an effective and strategic way to advance regional food policy and environmental change. A critical feature of the process was the ability of key policy actors, most notably Public Health and Planning, to *appeal* to, and align with, the interests and political sensitivities of decision makers. Viewed in this way, there was a close thematic overlap between “aligned agendas” and “issue framing” as sub-themes of “strategic positioning”.

##### Government Actors’ Concerns about “Legitimacy” (Sub-theme) in Food System Policy Making

Legitimacy refers to having an undisputed credibility with respect to action or position, and relates to the quality of being believable and trustworthy [17]. Within this case study, key informants reflected on their experience of regional food policy making and identified issues of concern regarding “legitimacy”. Specifically, comments related to the legitimacy of various stakeholders’ roles (including the role of government) and the ways in which stakeholders engage as valued players in regional food system activity (*i.e.*, production, distribution, retailing, *etc*. or policy making). The concept of “*legitimate participation*” emerged through participants’ descriptions of individuals and groups who had established credibility (e.g., knowledge and skills) or demonstrated trustworthiness or expertise on food system issues. As well, it also referred to those who had supported meaningful environmental change in the Region through the transfer and dissemination of innovative ideas or novel local food system practices. In most instances of “legitimate participation”, it was participants themselves who, by reflecting on personal experiences and those of others, constructed an answer to the question of “*Who can legitimately participate in regional food policy making *(*and other forms of food system activity*)?” Thus, an examination of the sub-theme of “legitimacy” captured the various ways in which participants defined, understood and communicated “*legitimate*” food system participation.

The Region’s public health and planning departments were most commonly the subject of discussion regarding “legitimate” food system participation. With respect to the role of Public Health, there were mixed perspectives on the perceived *legitimacy *of staff actions. For example, some participants questioned the Department’s motivation and investment in non-mandated activities while others saw staff as having a genuine concern for local food system stakeholders. From a public health perspective, staff were motivated by the need for supportive policy and environmental changes yet found it difficult to engage the community and attract the interest and participation of other government actors and regional departments. Public Health’s ability to participate effectively in food policy making was also affected by gaps in the Ontario Public Health Standards at the time and by a lack of regional support (*i.e.*, mandate and funding constraints for food system activity). However, by raising awareness of the issues affecting food access and farmer viability, it was acknowledged that Public Health was effectively helping to “*legitimize*” what was regarded by some as “fringe activity” by increasing government support and recognition of a number of inter-related food system concerns. Thus, based on multi-sectoral perspectives, Public Health established credibility by translating knowledge into practice, building community capacity, and marshaling internal departmental support for a food system agenda through a strategic partnership with Planning. 

“Legitimacy” was also discussed in regards to the role and motivations of the Region’s Planning Department. According to policy experts, Area Municipalities questioned the *legitimacy *of the Region’s involvement in food-related issues and opposed their early attempts to influence the size and location of food stores at the local level. Specifically, municipal planners argued that Planning was trying to influence commercial planning and *overstepping its jurisdictional authority* as a regional department. From a policy perspective, the challenges of “legitimately” navigating food access considerations as a way to implement supportive environmental changes (as a new area of policy and practice) are recognized by the following regional expert:

“*It*’*s a bit of a struggle to find wording that you can say*, *legitimately*, *in an official plan around these issues*. *We*’*re stepping into areas of jurisdiction over which some would question why we*’*re even involved*.”

(*Regional Policy Planner*, *2009*)

To minimize the tensions and challenges associated with their new role and interest in food policy making, government actors in Planning emphasized the importance of a collaborative and shared responsibility in promoting the regional food system*. *This was done by: (1) including both regional and municipal food planning directives in the ROP; (2) allowing flexibility in policy interpretation and implementation across area municipalities; and (3) developing a specific policy statement to acknowledge the ongoing, and necessary contributions of the Region’s Public Health Department to food system change (Policy 3.F.6 of the Regional Official Plan: “The Region will collaborate with stakeholders to continue to implement initiatives supporting the development of a strong regional food system”[7]). The latter was also acknowledged as a strategic and intentional way to increase the legitimacy of a regional government role in food policy and environmental change:

“*The policy is consistent with the work that Public Health was already doing*…*So we thought it was logical to mesh in with that and if anything, provide some support in our Plan for the work that they*’*re doing and to see if there was a way that we could have that work continue in the future*.”

(*Regional Policy Planner*, *2009*)

This example highlights the link between the overarching theme of “partnerships” and the sub-theme of “legitimacy” and suggests that supportive partnerships can help to increase the acceptability, or perceived “legitimacy” of new government roles. In contrast, on issues where Public Health and key stakeholders disagreed in their approach to addressing regional food system concerns (and subsequently “broke ties” or discontinued a community partnership), relational tensions, competition for funds and overlapping stakeholder mandates were identified as negative effects of government participation. A critical challenge to establishing a legitimate government role in food system change was captured by the following food system stakeholder perspective:

“*Everybody sees their own piece of the puzzle and you*’*ve got so many different funding organizations and champions of food systems*…*and at the end of the day*, *I see very little true collaboration*.”

(*Food System Stakeholder*, *2009*)

Thus, with respect to “what worked” in establishing “legitimate” government roles in food system policy making and food system change in Waterloo Region, it was found that those who were most effective in contributing to supportive food system actions were government actors who had: established a history of significant food system involvement (e.g., Public Health’s groundwork in “creating a climate of change” [14]); built a reputation for leadership and progressive ideas; operated within an appropriate mandate (despite adopting a new or non-traditional role); and collaborated in a manner that minimized threats, competition and tensions with other food system actors. Strategic departmental partnerships and collaboration between food system stakeholders were salient features of legitimacy in this context. Overall, it was found that partnerships and collaboration among stakeholders can increase one’s legitimate participation in food policy and environmental change while competing mandates and tensions can restrict, or limit the ability of legitimate stakeholders to participate in regional food system change.

## 4. Discussion and Conclusions

The overarching themes of “strategic positioning”, “partnerships” and “knowledge transfer” and the key underlying themes (sub-themes) of “aligned agendas”, “issue framing”, “visioning” and “legitimacy” emerged from multi-sectoral perspectives of the roles and motivations of government actors in food system policy making and environmental change. An exploration of these themes through relevant case study examples from Waterloo Region offers insight into the ways in which government actors can act to facilitate access to healthy food alongside other important, and sometimes, competing priorities. For some governments, greater attention and support for regional and local food systems may be an important way to advance a number of inter-related social, economic and environmental goals.

While the local and historical contexts in this case study limits the transferability of the findings beyond Waterloo Region, this study helped to address several important gaps in the literature concerning the ways in which food system planning “ideas” reach the political agenda, are considered by government, and become adopted as part of official land use policies. It was shown that government actors (particularly passionate local food and agriculture champions within government) can use “strategic positioning”, internal and external “partnerships” and “knowledge transfer” (and knowledge exchange) to disseminate new food ideas and policy options. As well, the “alignment” of political agendas, the use of “visioning” exercises to disseminate new ideas, and the strategic use of appropriate policy “frames” were discussed by 47 key informants as effective ways to engage in food policy making and regional food system change. While the context will differ in other jurisdictions, the identification of strategic assets, the value of strategizing across all levels of government and the use of internal and external partners to help advance supportive food policy considerations were shown as effective strategies (*i.e.*, “what worked”) in Waterloo Region and can similarly have meaningful application elsewhere. While the local and historical context was unique to Waterloo Region, the identification of key overarching themes and subthemes concerning the role and motivations of government in food system policy making were presented as a starting point to guide multi-sectoral dialogue and collaborative action in other jurisdictions. While Waterloo Region is the first regional municipality to legally prescribe food system policies within the comprehensive planning process, other jurisdictions and policy actors may be keen to consider the applicability of these findings to their efforts to address similar food system-related challenges. 

A socio-ecological framework [18] offers a way of understanding the multiple factors and influences that shape the eating behaviours of individuals and groups [6]. This case study explored the development of food policies and supportive planning practices as important upstream influences of physical access to food at the community level. In addition, Public Health’s capacity building and awareness raising activities and Planning’s use of “visioning” within the comprehensive planning process were identified as early and strategic approaches to influencing values, attitudes, beliefs and food norms within the Region’s socio-cultural environment. Overall, it was found that when this type of food system groundwork and awareness raising activities precede policy and planning decisions (policy adoption), there is greater political interest, public support, and potential for cross-sectoral collaboration to address community food security alongside other regional priorities. Specifically, improved access to healthy food was a key public health objective that aligned well with multi-sectoral interests in farmer viability, environmental and agricultural land protection, and urban intensification. Thus, while a socio-ecological framework is useful for understanding how upstream factors shape the environments within which individuals and groups make food-related decisions, a food *system* approach (or view of the problem) offered a complementary lens through which to examine the various points of intersection that influence how and why government actors are willing to engage in food system policy making and environmental change. 

These findings are consistent with earlier research on the environmental determinants of healthy eating [6,19,20] but offer rich insight into the roles and motivations of government actors in creating supportive policies and environments to facilitate access to healthy food. Specifically, the findings shed light on the various points of intersection that can be used to promote multi-sectoral dialogue and collaborative action to address various aspects of community food security at the regional level. It was shown that healthy public policies, and other supportive physical and social environmental changes to improve healthy food access could be achieved by finding ways (*i.e.*, points of intersection) to attract the interest and investment of multi-sectoral stakeholders. Social and environmental goals including healthier residents, fewer redundant imports of food produced locally, increased numbers of family farms, and agricultural land preservation were important motivations for government participation in regional policy and environmental change. In light of these findings, a conceptual framework is presented to illustrate the overarching themes, sub-themes and features of food system policy making and environmental change based on a case study of Waterloo Region. This framework can be used in other jurisdictions as a starting point to help engage and align the interests of multi-sectoral stakeholders towards plans and actions to support local and regional food systems.

**Figure 1:**
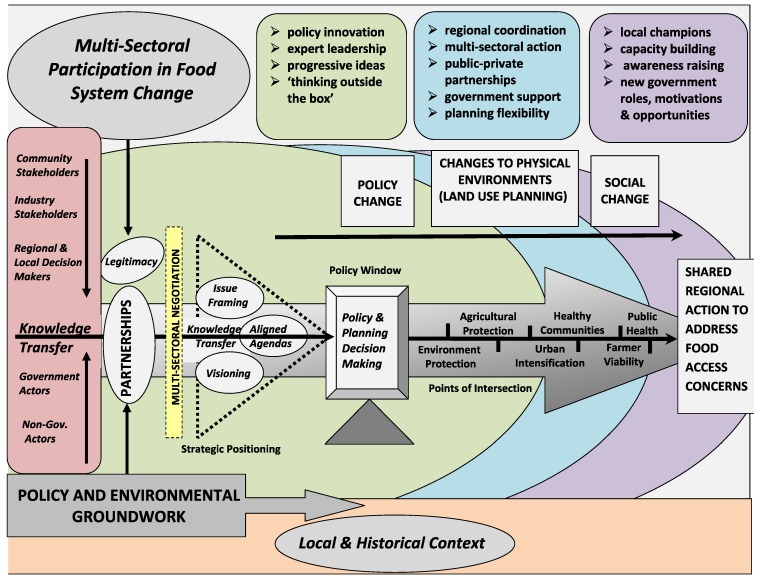
A conceptual framework for multi-sectoral participation and action in food system change.

The conceptual framework is presented in Figure 1 and incorporates a new view of coordinated and collaborative multi-sectoral action towards improving community food access alongside other regional priorities. The framework integrates the key overarching themes and sub-themes from this case study and identifies areas where leaders in public health and planning can work to create multi-sectoral partnerships to advance policy, and improve physical and social environments to facilitate and support access to healthy food.

Based on the socio-ecological model, this framework is limited to the community- and policy-level influences that shape food access and does not consider the interpersonal and organizational processes that are relevant influences of dietary behavior. The focus is on the way regional government and local actors organize to improve access to food alongside other regional priorities. A limitation of this framework is that it is based on a shared construction of participants’ lived experience in Waterloo Region and the researchers’ interpretation of that experience.

The conceptual framework presented in Figure 1 offers a theoretical foundation for further scholarly research by reducing the complexity of food policy making activity in Waterloo Region into key themes that can be explored in other settings, policy contexts, and regional municipalities. Although significant within Waterloo Region, overarching themes (strategic positioning, partnerships, and knowledge transfer) and subthemes (agenda alignment, visioning, issue framing and legitimacy) will need to be evaluated for their transferability and applicability in other jurisdictions.

The research served to answer an important and timely question concerning “*what works*?” with respect to the role of government in food policy making and food system change. At the time of this study, many government and non-government groups are engaged in work on various platforms and positions to address food security in Canada [21,22,23,24]. Although little progress has been made nationally, key findings from this research regarding the need for strategic positioning, partnerships and aligned political agendas may offer insight for food policy considerations at provincial and federal levels. From a public health perspective, supportive action can help drive change and promote positive improvements in community food security. With committed government support and regional coordination, various local food system initiatives could be promoted to increase access to healthy, local food and contribute to important gains in population health over time.
